# Impact of scaling up health intervention coverage on reducing maternal mortality in 26 low- and middle-income countries: A modelling study

**DOI:** 10.7189/jogh.14.04221

**Published:** 2024-11-22

**Authors:** Xi-Ru Guo, Yue-Long Ji, Shi-Yu Yan, Ting Shi, Kanittha Chamroonsawasdi, Jue Liu, Hai-Jun Wang

**Affiliations:** 1 Department of Maternal and Child Health, School of Public Health, Peking University, National Health Commission Key Laboratory of Reproductive Health, Peking University Health Science Center-Weifang Joint Research Center for Maternal and Child Health, Beijing, China; 2 Usher Institute, University of Edinburgh, Edinburgh, UK; 3 Department of Family Health, Faculty of Public Health, Mahidol University, Bangkok, Thailand; 4 Department of Epidemiology and Biostatistics, School of Public Health, Peking University, Beijing, China

## Abstract

**Background:**

Prioritising actions is urgently needed to address the stagnation of the global maternal mortality ratio (MMR). As most maternal deaths occur in low- and middle-income countries (LMICs), we aimed to assess the impact of scaling up health intervention coverage on reducing MMR under four scenarios for 26 LMICs.

**Methods:**

We conducted a modelling study to estimate the MMR and additional maternal lives saved by intervention by 2030 for 26 LMICs using the Lives Saved Tool (LiST). We used four scenarios to assess the impact of scaling up health intervention coverage by no scale-up (no change), modest scale-up (increased by 2% per year), substantial scale-up (increased by 5% per year), and universal coverage (coverage reached 95% by 2030). We divided the selected 26 countries into three groups according to their MMR levels in 2020.

**Results:**

Among 26 LMICs, six (23.1%) countries showed an increase in MMRs and 13 (50.0%) stalled on the reduction of MMR from 2015 to 2020. Under a substantial scale-up of coverage or scaling up to universal coverage, the average MMR in 2030 of 26 LMICs would be 62.8 or 52.8, reaching the Sustainable Development Goal (SDG) 3.1. Caesarean delivery, uterotonics for postpartum haemorrhage, and assisted vaginal delivery had a more important role in this reduction compared to other interventions.

**Conclusions:**

Scaling up the coverage of health interventions is critical for reducing MMRs. If a substantial scale-up or scaling up to universal coverage of continuous maternity interventions from preconception to postpartum period can be achieved, LMICs in Southeast Asia and Western Pacific regions could reach the SDG 3.1 on time.

Women’s survival, health, and well-being are among the focusses for achieving Sustainable Development Goals (SDGs) [1], with SDG target 3.1 being to reduce the global maternal mortality ratio (MMR) to less than 70 maternal deaths per 100 000 live births by 2030, with no country having an MMR greater than 140 per 100 000 live births. According to MMR estimates for 2020 by the World Health Organization (WHO), United Nations International Children’s Emergency Fund (UNICEF), United Nations Population Fund (UNFPA), World Bank Group, and United Nations Department of Economic and Social Affairs (UNDESA)/Population Division, the global MMR was 223 maternal deaths per 100 000 live births in 2020 and about 287 000 women died during and following pregnancy and childbirth in 2020 in the world [2]. In 2020, 95% of maternal deaths were clustered among low- and middle-income countries (LMICs) [2,3]. Despite advancements in reducing global maternal mortality in the past two decades, further and sustained efforts are necessary to reduce maternal deaths and end preventable maternal mortality in such contexts.

In 2020, the average MMR in LMICs within the WHO Southeast Asia and Western Pacific regions was 99 per 100 000 live births [2]. Since 2015, Southeast Asia and Western Pacific regions have experienced a concerning stagnation, with some countries even demonstrating a reversal in progress in achieving the MMR target [2,4]. Prioritising actions is urgently needed to reduce MMR for achieving SDG target 3.1 on time. Scaling up the coverage of life-saving evidence-based health interventions is essential for reducing preventable maternal mortality [5]. Quantifying the impact of health intervention coverage on maternal mortality reduction by intervention at national and regional levels could provide evidence for optimising the priority of health system investment in maternal health interventions. One study using data from 1990–2008 focussed on the Association of Southeast Asian Nations (ASEAN) to illustrate the impact of intervention coverage on maternal mortality [6], while another showed the effect of midwives on maternal mortality in LMICs [7]. However, there is a lack of evidence on the impact of scaling up continuous maternity intervention coverage on reducing maternal mortality in LMICs in Southeast Asia and Western Pacific regions after 2015.

To support country-level and region-level decision-making about health system investments for LMICs, we aimed to estimate the impact of health interventions on reducing maternal mortality in the selected 26 LMICs from Southeast Asia and Western Pacific regions under four intervention coverage scenarios. We estimated MMR and additional maternal lives saved by intervention under various scenarios for scaling up the coverage of health interventions by 2030. Our findings could help health planners in LMICs within these regions to assess and expand effective interventions that address target needs for reducing MMR.

## METHODS

### Overview

In this modelling study, we used the Lives Saved Tool (LiST) embedded within the Spectrum software suite, version 6.29 (Avenir Health, Sunnyvale, California, US) to model the impact of scaling up health interventions on maternal mortality. Spectrum software included the best available estimates of baseline health status and population size [8], while LiST’s default assumptions included the efficacy and affected fraction of interventions on cause-specific maternal mortality (Table S1 in the **Online Supplementary Document**). We used all the default information in this study except for baseline maternal mortality data. We updated the default baseline MMR data to MMR estimates for 2020 according to the sources from WHO, UNICEF, UNFPA, World Bank Group, and UNDESA/Population Division [2]. We updated the default baseline percent of maternal deaths by using causes data of maternal deaths for women aged 15–49 years from Global Burden of Disease (GBD) 2019. Detailed data sources and methodologies used in GBD 2019 have been reported elsewhere [9].

### Maternal mortality

In this study, we presented country-specific estimates of MMR in 2000–20 from the WHO, UNICEF, UNFPA, World Bank Group, and UNDESA/Population Division. To identify possible points when a change in the linear slope of the temporal trend of MMR occurred, we carried out joinpoint regression models considering data from 2000–20 for each country in 26 LMICs. We estimated an annual percentage change (APC) for each identified trend and used the average APC (AAPC) to characterise the overall rate of change.

We extracted causes of maternal deaths for women aged 15–49 years from the GBD 2019 study. According to a WHO systematic analysis of global causes of maternal death [10], we divided maternal haemorrhage into antepartum, intrapartum, and postpartum haemorrhage, which, according to our estimates, accounted for 24%, 3%, and 73% cases of maternal haemorrhage, respectively. The causes of maternal deaths in this study included antepartum haemorrhage, intrapartum haemorrhage, postpartum haemorrhage, hypertensive disorders, sepsis, abortion, and other direct and indirect causes.

### Scenarios

According to previous studies [7], we designed our analysis to show the potential impacts of increasing health intervention coverage in four scenarios. With no scale-up (scenario 0), we assumed that coverage of every health intervention did not change from 2020 (**Table 1**). With a modest scale-up (scenario 1), we assumed that coverage of every health intervention increased by 2% per year up to a maximum of 100%. With a substantial scale-up (scenario 2), we assumed that coverage of every health intervention increased by 5% per year up to a maximum of 100%. With universal coverage (scenario 3), we assumed that coverage of every health intervention reached 95% by 2030. We performed the sensitivity analysis by calculating the MMR in 2030 from the APC of 2015–20, where with the current trend (scenario 0), we assumed that coverage of every health intervention did not change from 2020 with MMR changing according to APC of 2015–20.

**Table 1:** Scenarios used to model the impact of health interventions on maternal mortality

Scenario	Description	Percentage change in intervention coverage rates
0	No scale-up	No change from the baseline (2020) coverage rates
1	Modest scale-up	2% increase in baseline coverage rates up to a maximum of 100%
2	Substantial scale-up	5% increase in baseline coverage rates up to a maximum of 100%
3	Universal coverage	95% coverage by 2030

### Estimating MMR and additional maternal lives saved

We used LiST to estimate MMR and additional maternal lives saved under each scenario. Considering the substantial uncertainty inherent in MMR, we computed the 80% uncertainty intervals (UIs), i.e. the10th and 90th percentiles of the posterior distributions, for MMRs in 2020 using their standard errors, which we then used to calculate the 80% UI for the estimated MMR in 2030.

As LiST separated results by intervention, we included health interventions with available and clear data sources. In this study, the continuum of maternity care, focussing on health interventions in each period of maternity health, consisted of one periconceptual intervention, three pregnancy interventions, and ten childbirth interventions. Periconceptual intervention was safe abortion services. Pregnancy interventions were tetanus toxoid vaccination, micronutrient supplementation (i.e. iron and multiple micronutrients) and hypertensive disorder case management. Childbirth interventions were a clean birth environment, magnesium sulphate for eclampsia, antibiotics for preterm or prolonged premature rupture of membranes, antibiotics for maternal sepsis, assisted vaginal delivery, uterotonics for postpartum haemorrhage, manual removal of placenta, removal of retained products of conception, caesarean delivery, and blood transfusion.

We included 26 LMICs in Southeast Asia and Western Pacific regions, excluding seven countries with a population of less than 100 000. Moreover, considering that SDG 3.1 is to reduce the global MMR to less than 70, with no country having an MMR greater than 140, we classified these 26 selected countries into three groups according to their MMR in 2020. Group A included LMICs with an MMR higher than 140 maternal deaths per 100 000 live births (Cambodia, Indonesia, Myanmar, Nepal, Papua New Guinea, and Timor Leste). Group B included LMICs with an MMR in 2020 from 70 to 140 maternal deaths per 100 000 live births (Bangladesh, Federated States of Micronesia, India, Kiribati, Laos, North Korea, the Philippines, Solomon Islands, Tonga, and Vanuatu). Group C included LMICs with an MMR of less than 70 maternal deaths per 100 000 live births (Bhutan, China, Fiji, Malaysia, Maldives, Mongolia, Samoa, Sri Lanka, Thailand, and Vietnam).

## RESULTS

### Maternal mortality and causes of death

Among 26 included LMICs, six (23.08%) countries (Cambodia, Federated States of Micronesia, Tonga, Vanuatu, Maldives, and Samoa) showed an increase in their MMR, while thirteen (50.00%) saw stagnation since 2015 (i.e. had a decline rate of MMR after 2015 less than the decline rate before 2015). Groups A, B, and C had an average of 190, 103, and 40 maternal deaths per 100 000 live births in 2030, respectively (**Table 2**).

**Table 2:** MMR and APC, AAPC of MMR in 2000–20 among 26 LMICs, stratified by group

	MMR					APC			
	**2000**	**2005**	**2010**	**2015**	**2020**	**2000–10**	**2010–15**	**2015–20**	**AAPC**
**Group A**	474	376	300	232	190	−4.44	−4.68	−4.07	−4.47
Cambodia	606	382	276	209	218	−8.92	−5.91	1.10	−4.98
Indonesia	299	276	219	194	173	−1.80	−3.65	−1.67	−2.70
Myanmar	371	321	293	243	179	−2.59	−2.65	−6.37	−3.58
Nepal	504	380	349	252	174	−5.00	−3.39	−8.83	−5.18
Papua New Guinea	312	314	289	208	192	1.02	−4.05	−2.43	−2.40
Timor Leste	750	584	376	285	204	−5.47	−6.93	−5.87	−6.30
**Group B**	225	187	151	122	103	−3.36	−4.18	−3.33	−3.83
Bangladesh	441	376	301	212	123	−2.73	−5.52	−10.78	−6.18
Federated States of Micronesia	60	57	46	64	74	−2.53	0.87	5.14	1.05
India	384	286	179	128	103	−6.35	−7.46	−4.17	−6.37
Kiribati	116	118	131	121	76	0.82	0.41	−9.59	−2.09
Laos	579	442	284	184	126	-5.28	−8.40	−7.26	−7.34
North Korea	186	122	130	108	107	−7.47	−1.03	−1.21	−2.73
Philippines	129	122	105	88	78	−1.04	−3.19	−2.50	−2.48
Solomon Islands	150	153	147	141	122	0.40	−0.81	−2.86	−1.03
Tonga	94	95	93	86	126	0.37	−0.96	7.71	1.48
Vanuatu	109	98	93	92	94	−2.21	−0.65	0.59	−0.74
**Group C**	100	69	54	44	40	−7.25	−4.43	−1.71	−4.48
Bhutan	305	186	117	74	60	−9.43	−8.81	−4.09	−7.81
China	58	46	33	26	23	−4.83	−5.56	−2.07	−4.52
Fiji	49	45	42	39	38	−1.68	−1.41	−0.56	−1.26
Malaysia	40	32	25	22	21	−4.49	−4.03	−0.06	−3.17
Maldives	114	79	60	57	57	−7.79	−3.24	0.84	−3.41
Mongolia	158	94	65	47	39	−9.96	−6.77	−3.40	−6.76
Samoa	75	62	62	58	59	−3.55	−0.62	0.07	−1.19
Sri Lanka	61	44	37	30	29	−6.24	−3.74	−0.80	−3.65
Thailand	48	40	35	30	29	−3.49	−2.86	−0.72	−2.49
Vietnam	88	66	60	52	46	−5.44	−2.35	−2.60	−3.19

APC – annual percentage change, AAPC – average APC, LMICs – low- and middle-income countries, MRR – maternal mortality ratio

Postpartum haemorrhage, hypertensive disorders, and abortion were the leading causes of maternal deaths in 26 LMICs (Table S2 in the **Online Supplementary Document**). The proportion of postpartum haemorrhage and hypertensive disorders gradually decreased with the decline of MMR. In group A, postpartum haemorrhage, hypertensive disorders, and abortion accounted for 19.68%, 16.51%, and 9.74% of cases in group A, 14.76%, 13.18%, and 10.00% of cases in group B, and 13.49%, 12.70%, and 4.9% cases in group C, respectively.

### Estimated MMR and additional maternal lives saved by 2030

**Figure 1:**
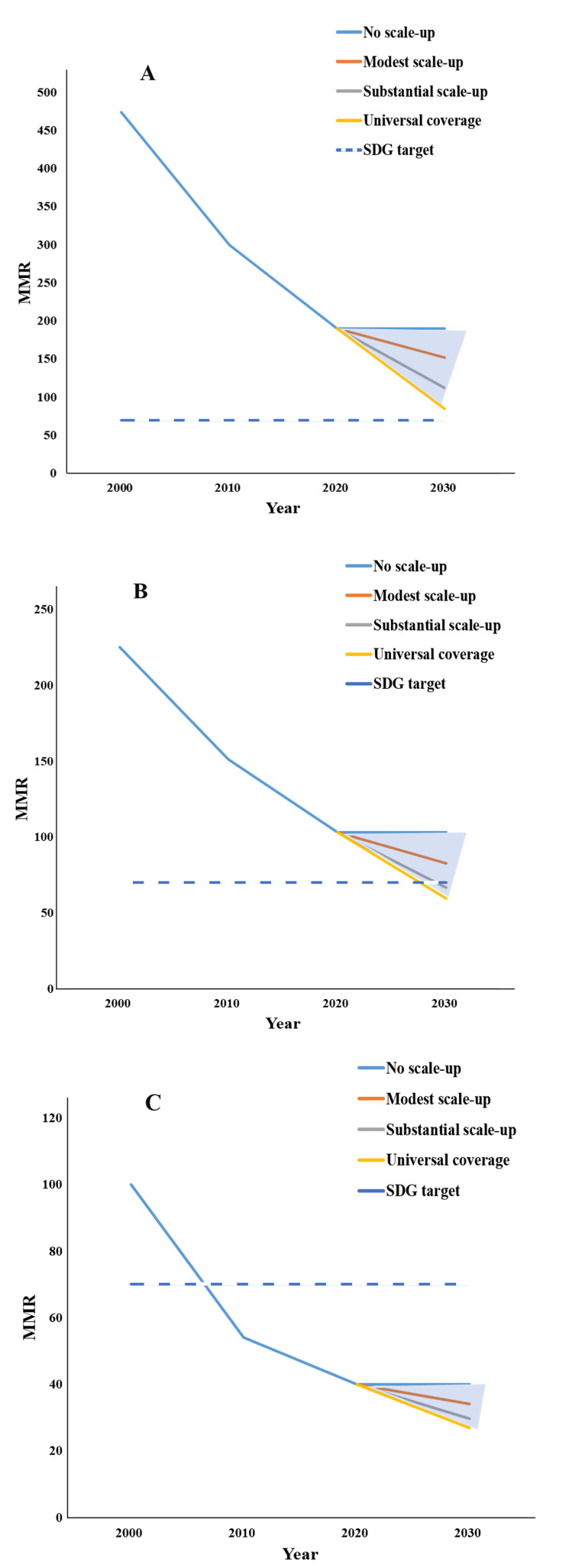
MMR under four scenarios among 26 LMICs in 2000–30. **Panel A.** Group A. **Panel B.** Group B. **Panel C.** Group C. MMR – maternal mortality ratio.

We estimated that, relative to their baseline status in 2020, under a substantial scale-up of coverage or scaling up to universal coverage, the average MMR in 2030 of LMICs in group A would be 112.03 or 84.90, averting 40.92% or 54.79% of maternal deaths; the average MMR in 2030 of LMICs in group B would be 66.43 or 59.41, averting 36.20% or 42.84% of maternal deaths. Under a substantial scale-up of coverage or scaling up to universal coverage, LMICs in group A could have an average MMR in 2030 of less than 140, while those in group B could have an average MMR in 2030 of less than 70, reaching SDG 3.1 (**Figure 1**, **Table 3**). In sensitivity analysis, with the current trend, we assumed that MMR from 2020 to 2030 changed according to APC of 2015–20 with the coverage of every health intervention not changing. Relative to status in 2030 with the current trend, under a substantial scale-up of coverage or scaling up to universal coverage, the average MMR in 2030 of LMICs in group A would mean the aversion of 32.42% or 47.77% of maternal deaths; the average MMR in 2030 of LMICs in group B would mean the aversion of 16.27% or 25.61% of maternal deaths (Table S3 in the **Online Supplementary Document**).

**Table 3:** Estimated relative reductions of MMR by 2030 in four scenarios among 26 LMICs, stratified by group

	Scenario 0	Scenario 1		Scenario 2		Scenario 3	
	**MMR (80% UI)**	**MMR (80% UI)**	**Reduction, %**	**MMR (80% UI)**	**Reduction, %**	**MMR (80% UI)**	**Reduction, %**
**Total**	99 (72, 174)	79.96 (53.18, 129.22)	19.11	62.82 (41.67, 101.89)	36.44	52.78 (33.87, 86.19)	26.63
**Group A**	190 (160, 348)	152.24 (106.86, 232.26)	20.00	112.03 (78.60, 171.21)	40.92	84.90 (55.58, 130.31)	54.79
Cambodia	218 (156, 326)	178.96 (128.07, 267.62)	17.91	123.11 (88.10, 184.10)	43.53	83.63 (59.84, 125.06)	61.64
Indonesia	173 (121, 271)	129.94 (90.88, 203.55)	24.89	91.24 (63.81, 142.92)	47.26	76.33 (53.39, 119.57)	55.88
Myanmar	179 (125, 292)	132.75 (92.70, 216.55)	25.84	96.42 (67.33, 157.29)	46.13	63.58 (44.40, 103.72)	64.48
Nepal	174 (125, 276)	148.72 (106.84, 235.90)	14.53	124.28 (89.28, 197.13)	28.57	122.57 (88.05, 194.42)	29.56
Papua New Guinea	192 (126, 293)	156.98 (103.02, 239.56)	18.24	121.41 (79.68, 185.28)	36.77	90.90 (59.65, 138.72)	52.66
Timor Leste	204 (147, 283)	166.06 (119.66, 230.37)	18.60	115.72 (83.39, 160.54)	43.27	72.36 (52.14, 100.38)	64.53
**Group B**	103 (63, 183)	82.47 (50.25, 145.93)	20.34	66.43 (40.30, 118.19)	36.20	59.41 (35.78, 106.17)	42.84
Bangladesh	123 (89, 174)	107.11 (77.50, 151.52)	12.92	90.87 (65.75, 128.55)	26.12	79.47 (57.50, 112.42)	35.39
Federated States of Micronesia	74 (32, 169)	60.50 (26.16, 138.16)	18.24	50.65 (21.90, 115.67)	31.55	45.98 (19.88, 105.01)	37.86
India	103 (93, 110)	81.89 (73.94, 87.46)	20.50	64.32 (58.07, 68.69)	37.55	55.28 (49.91, 59.04)	46.33
Kiribati	76 (33, 146)	59.82 (25.98, 114.92)	21.29	49.68 (21.57, 95.43)	34.63	43.48 (18.88, 83.53)	42.79
Laos	126 (92, 185)	109.79 (80.16, 161.20)	12.87	89.69 (65.49, 131.69)	28.82	75.66 (55.24, 111.09)	39.95
North Korea	107 (46, 249)	87.96 (37.81, 204.69)	17.79	68.42 (29.69, 160.71)	36.06	60.35 (25.94, 140.43)	43.60
Philippines	78 (67, 96)	58.39 (50.15, 71.86)	25.14	39.72 (34.12, 48.89)	49.08	36.81 (31.67, 45.30)	52.81
Solomon Islands	122 (75, 197)	91.44 (56.21, 147.65)	25.05	72.46 (44.54, 117.00)	40.61	66.14 (40.66, 106.80)	45.79
Tonga	126 (55, 289)	104.33 (45.54, 239.30)	17.20	91.89 (40.11, 210.76)	27.07	85.74 (37.42, 196.65)	31.95
Vanuatu	94 (43, 211)	63.50 (29.05, 142.55)	32.45	46.55 (21.79, 104.49)	50.48	45.21 (20.68, 101.47)	51.90
**Group C**	40 (28, 60)	34.07 (23.90, 50.68)	14.36	29.70 (20.89, 44.00)	24.94	26.88 (18.94, 39.74)	32.15
Bhutan	60 (40, 82)	52.02 (34.68, 71.09)	13.30	44.49 (29.66, 60.80)	25.85	37.94 (25.30, 51.86)	36.77
China	23 (19, 27)	20.98 (17.33, 24.63)	8.78	19.41 (16.03, 22.78)	15.61	17.89 (14.78, 21.00)	22.22
Fiji	38 (28, 55)	31.39 (23.13, 45.43)	17.39	27.29 (20.11, 39.50)	28.18	23.96 (17.65, 34.68)	36.95
Malaysia	21 (18, 29)	18.78 (16.10, 25.94)	10.57	16.76 (14.37, 23.15)	20.19	14.91 (12.78, 20.59)	29.00
Maldives	57 (40, 83)	49.01 (34.39, 71.37)	14.02	43.23 (30.34, 62.95)	24.16	43.05 (30.21, 62.69)	24.47
Mongolia	39 (28, 55)	32.31 (23.20, 45.57)	17.15	28.98 (20.81, 40.87)	25.69	30.29 (21.74, 42.71)	22.33
Samoa	59 (26, 137)	47.45 (20.91, 110.19)	19.58	39.67 (17.48, 92.13)	32.76	34.33 (15.13, 79.71)	41.81
Sri Lanka	29 (24, 38)	25.20 (20.86, 33.03)	13.10	21.57 (17.85, 28.26)	25.62	17.93 (14.84, 23.49)	38.17
Thailand	29 (24, 34)	25.24 (20.89, 29.59)	12.97	21.92 (18.14, 25.70)	24.41	19.45 (16.09, 22.80)	32.93
Vietnam	46 (33, 60)	38.32 (27.49, 49.98)	16.70	33.63 (24.13, 43.87)	26.89	29.05 (20.84, 37.89)	36.85

**Figure 2:**
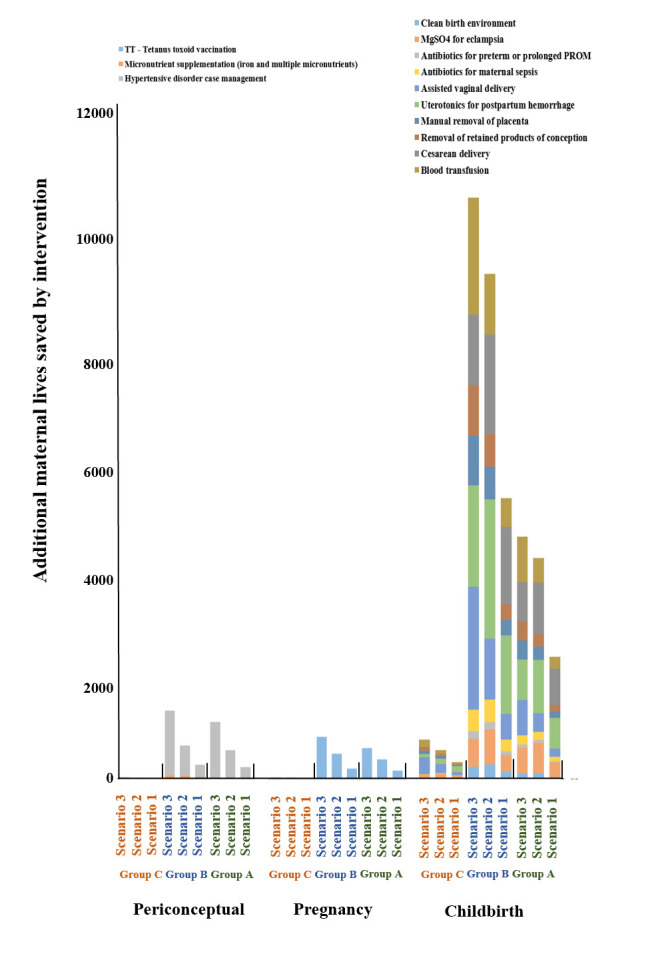
Additional maternal lives saved by intervention under three scenarios among 26 LMICs in 2030.

In LMICs in group A, additional maternal lives saved by scaling up the coverage of health interventions were 2585 in scenario 1, 4906 in scenario 2, and 6019 in scenario 3. Caesarean delivery played a more important role than other interventions, accounting for 25.88% in scenario 1, 19.06% in scenario 2, and 11.80% in scenario 3 of the total additional maternal lives saved. In LMICs in group B, additional maternal lives saved by scaling up the coverage of health interventions were 5566 in scenario 1, 10 285 in scenario 2, and 12 617 in scenario 3. Uterotonics for postpartum haemorrhage played a more important role compared to other interventions, accounting for 25.75% in scenario 1, 24.74% in scenario 2, and 14.73% in scenario 3 of the total. In LMICs in group C, additional maternal lives saved by scaling up the coverage of health interventions were 315 in scenario 1, 550 in scenario 2, and 768 in scenario 3. Assisted vaginal delivery played a more important role than other interventions, accounting for 20.00% in scenario 1, 28.91% in scenario 2, and 40.49% in scenario 3 of the total (**Figure 2**; Table S4 in the **Online Supplementary Document**). In LMICs in group A, every country could reduce MMR to less than 140 under a substantial scale-up or scaling up to universal coverage. In LMICs in group B, the Federated States of Micronesia, India, Kiribati, North Korea, the Philippines, and Vanuatu could reduce MMR to less than 70 under a substantial scale-up of coverage or scaling up to universal coverage (**Table 3**).

In LMICs in group A, maternal lives saved by scaling up the coverage of childbirth interventions were 2224 (86.03% of the total) in scenario 1, 4028 (82.10% of the total) in scenario 2, and 4420 (73.43% of the total) in scenario 3 (Table S4 in the **Online Supplementary Document**). In LMICs in group B, maternal lives saved by scaling up the coverage of childbirth intervention were 5123 (92.04% of the total) in scenario 1, 9220 (89.65% of the total) in scenario 2, and 10 613 (84.12% of the total) in scenario 3. In LMICs in group C, maternal lives saved by scaling up the coverage of childbirth interventions were 300 (95.24% of the total) in scenario 1, 519 (94.36% of the total) in scenario 2, and 714 (92.97% of the total) in scenario 3. We further assumed that coverage of childbirth health interventions increased by 5% per year up to a maximum of 100%. Under this scenario, the average MMR of LMICs in group B would be 73.67, not reaching the SDG 3.1 (Table S5 in the **Online Supplementary Document**).

## DISCUSSION

To our knowledge, this modelling study was the first regional maternal mortality study focussing on LMICs in Southeast Asia and Western Pacific regions. We found that the reduction of MMR in LMICs has stagnated since 2015, with some countries even increasing in MMR. We also found that relative to baseline status in 2020, under a substantial scale-up of coverage or scaling up to universal coverage, the average MMR in 2030 of LMICs in group A would be 112.03 or 84.90, while the average MMR in 2030 of those in group B would be 66.43 or 59.41. These findings indicated that a substantial scale-up or scaling up to universal coverage of continuous maternity interventions from preconception to postpartum period could help LMICs in Southeast Asia and Western Pacific regions reach the SDG target 3.1 on time.

Maternal mortality serves as a key metric for assessing health care quality and shaping policy initiatives, as it encapsulates not only the direct fatalities, but also underlying systemic issues and disparities that impact maternal well-being [11,12]. Six (23.08%) countries – Cambodia, Federated States of Micronesia, Tonga, Vanuatu, Maldives and Samoa – saw an increase in their MMR, while half (50.00%) saw a stagnation in their previously decreasing trends since 2015. Heightened attention and intensified efforts are needed to address this change, end preventable maternal mortality, and achieve SDG target 3.1 for LMICs in these regions. We also saw that for LMICs in groups A, B, and C, the average MMRs in 2030 were 190, 103, and 40, respectively. This inequality of maternal deaths in the selected 26 countries necessitates serious consideration and enough attention.

A 2014 WHO systematic analysis suggested that, except for other direct causes and indirect causes, haemorrhage was the leading cause of maternal death, followed by hypertensive disorders and sepsis [10]. We found that, except for other direct causes and indirect causes, postpartum haemorrhage, hypertensive disorders, and abortion were the three leading causes of maternal deaths in 26 LMICs. Additionally, with the decline of the level of MMR, the proportion of postpartum haemorrhage and hypertensive disorders gradually decreased. Notably, we found the transition in the proportion of causes of death and the significance of abortion.

Reducing maternal mortality is a multifaceted challenge stemming from multiple factors. Based on the three delays model from a prior study [13], maternal mortality depended on the delay in decision-making due to insufficient knowledge and low awareness of the pregnant women and their family members, delays in reaching care caused by poor economic status and remote living conditions, and delays in receiving adequate care due to a lack of manpower, drugs, and poor management. Recommendations to reduce maternal mortality include decreasing fertility rates (particularly among adolescents), increasing per-capita health spending, reducing out-of-pocket health care expenses, and improving coverage of health interventions, all of which have proven effective [14]. Among them, expanding health intervention coverage was the most important element [3]. Most efficient maternal health interventions have not yet achieved universal coverage in Southeast Asia and Western Pacific regions [15]. Furthermore, a prior study found that inequities in accessing effective maternal health interventions persisted across Southeast Asia and the Western Pacific regions [16]. Health interventions included in our modelling study were aligned with the reproductive, maternal, newborn, and child health services mentioned in the universal health coverage target [17]. Scaling up maternal health intervention coverage was a joint step towards realising both the SDGs and universal health coverage.

We found that health investment in comprehensive emergency care (especially caesarean delivery) and basic emergency care (especially uterotonics for postpartum haemorrhage and assisted vaginal delivery) could be increased and given priority attention for LMICs in Southeast Asia and Western Pacific regions. Different interventions were previously found to have different effects on reducing different causes of maternal mortality [18]. Uterotonics is one of the important preventions for postpartum haemorrhage recommended by the WHO [19], while caesarean delivery serves as a lifesaving intervention for numerous emergency obstetrical, such as obstructed labour [20]. Assisted vaginal delivery, meanwhile, is important in preventing maternal mortality and offers both paediatric and maternal beneﬁts of vaginal birth, which are not provided by caesarean deliveries [21]. Apart from emergency obstetric care, health interventions in preconception and pregnancy are also critical. According to a previous study [22], an effective provision of a continuum of care in maternal health services for women can avert 113 000 maternal deaths annually.

In this study, we estimated that under a substantial scale-up of coverage or scaling up to universal coverage, LMICs in group A could have an average MMR in 2030 of less than 140, while those in group B have an average MMR in 2030 of less than 70, reaching SDG 3.1. A substantial scale-up or scaling up to universal coverage of maternal health interventions is vital for LMICs to reduce MMR and achieve the SDG target 3.1 on time. For LMICs where MMR remained at a high level and government resources were limited, such as Myanmar, Cambodia, Nepal, and Indonesia, they could focus on increasing the coverage of prioritising interventions related to emergency obstetric care [23–28]. However, scaling up the coverage of specific important interventions (e.g. caesarean section) is not always better [29]. Therefore, the timing and quality of interventions need to be carefully considered. For example, increasing the coverage of uterotonics for postpartum haemorrhage and caesarean delivery by 5% per year could reduce Indonesia’s MMR to 131.78 in 2030. Similarly, increasing the coverage of caesarean delivery and safe abortion services by 5% per year could lower Myanmar’s MMR to 138.93 in 2030 (Table S6 in the **Online Supplementary Document**). For LMICs where MMR did not reach SDG 3.1, but had favourable political climates, such as India and the Philippines, they could pay more attention to the continuum of maternity interventions from preconception to the postpartum period, which was more feasible and cost-effective [30,31]. Good quality care in preconception and pregnancy could effectively prevent postpartum haemorrhage and other direct causes of maternal death in the early stage. We also found that for LMICs in group B, the average MMR in 2030 would not achieve SDG 3.1 by only increasing the coverage of childbirth interventions substantially. Simultaneously, the Maternal Death Surveillance and Response System should be established and strengthened as a nationwide program to monitor maternal mortality and timely plan to manage the problem. For LMICs with a low level of MMR, such as China and Thailand, efforts could be made to increase the coverage and quality of new cost-effective maternal interventions and reduce urban vs. rural and regional disparities [32,33]. Health cooperation in the 26 LMICs we selected here has already existed in Southeast Asia and the Western Pacific regions; one such example is the Belt and Road Initiative and Healthy Silk Road [34]. These LMICs could share their experience in health governance and enhance cultural exchanges to reduce health inequality and end preventable maternal mortality.

This study has several limitations. First, there are bound to be discrepancies between the estimates of our model and the real world. However, previous studies have shown the robustness of the default assumptions in the LiST model in terms of baseline health status, population size, and intervention effectiveness [7,8]. Second, our study focussed on the impacts of health intervention coverage on reducing maternal mortality, while the LiST model also took effectiveness, quality, and use of health interventions into account. Third, while we did use the 2020 MMR data as the baseline for our analysis due to delays in data reporting, we should note that this data is comprehensive, up-to-date, and accounted for the potential impact of COVID-19. Lastly, half of the selected 26 LMICs were small countries with a population of less than 10 million, presenting a higher risk of calculation errors. However, our results remained robust after excluding these 13 countries (Table S7 in the **Online Supplementary Document**).

## CONCLUSIONS

The reduction of MMR in LMICs has stalled since 2015, with some countries even experiencing an increase. Our findings highlight that LMICs with high MMR and limited government resources should focus on increasing the coverage of priority emergency obstetric care interventions. For LMICs where MMR did not reach SDG 3.1, but had favourable political climates, efforts should be directed towards significantly scaling up universal coverage of continuous maternity interventions from preconception to the postpartum period. Lastly, LMICs with a low MMR should focus on improving the coverage and quality of new, cost-effective maternal interventions. Taken as a whole, our results provide evidence-based guidance for shaping national and regional health policies, as well as determining the intensity and priority of health investments needed to reach SDG 3.1 and eliminate preventable maternal deaths in Southeast Asia and Western Pacific regions.

## Additional material


Online Supplementary Document
