# Supplementary material for: Impact of scaling up health intervention coverage on reducing maternal mortality in 26 low- and middle-income countries: A modelling study
Source: J Glob Health. 2024 Nov 22;14:04221. doi: 10.7189/jogh.14.04221 (PMC11583284; doi:10.7189/jogh.14.04221)
Supplement: Online Supplementary Document [file jogh-14-04221-s001.pdf]

Table S1 Default efficacy and affected factor in the LiST (Lives Saved Tool)

Causes of maternal death	Intervention	Efficacy	Affected fraction
**Intrapartum hemorrhage**			
	Blood transfusion	0.50	1.00
**Postpartum hemorrhage**			
	Uterotonics for postpartum hemorrhage	0.78	1.00
	Manual removal of placenta	0.30	1.00
	Removal of retained products of conception	0.30	1.00
	Blood transfusion	0.50	1.00
**Hypertensive disorders**			
	Hypertensive disorder case management	0.50	1.00
	$MgSO_4$ for eclampsia	0.59	1.00
	Cesarean delivery	0.45	1.00
**Sepsis**			
	Clean birth environment	0.60	1.00
	Antibiotics for preterm or prolonged PROM	0.80	0.33
	Antibiotics for maternal sepsis	0.80	1.00
	Blood transfusion	0.25	1.00
**Abortion**			
	Safe abortion services	0.95	0.91
**Other direct causes**			
	Assisted vaginal delivery	0.39	1.00
	Cesarean delivery	0.90	0.50
**Indirect causes**			
	TT - Tetanus toxoid vaccination	0.98	0.00

Table S2 Percent of maternal death in 2020 by cause by group among 26 LMICs

	Group A (%)	Group B (%)	Group C (%)
**Antepartum hemorrhage**	6.49	4.87	4.45
**Intrapartum hemorrhage**	0.90	0.67	0.62
**Postpartum hemorrhage**	19.68	14.76	13.49
**Hypertensive disorders**	16.51	13.18	12.70
**Sepsis**	7.26	8.47	3.63
**Abortion**	9.74	10.00	4.98
**Other direct causes**	26.48	37.27	43.20
**Indirect causes**	12.94	10.78	16.92

Notes: Group A (MMR higher than 140 maternal deaths per 100,000 live births) included Cambodia, Indonesia, Myanmar, Nepal, Papua New Guinea, and Timor Leste. Group B (MMR from 70 to 140 maternal deaths per 100,000 live births) included Bangladesh, North Korea, India, Kiribati, Laos, Federated States of Micronesia, the Philippines, Solomon Islands, Tonga and Vanuatu. Group C (MMR less than 70 maternal deaths per 100,000 live births) included Bhutan, China, Fiji, Malaysia, Maldives, Mongolia, Samoa, Sri Lanka, Thailand and Viet Nam.

Table S3 Estimated relative reductions on MMR by 2030 in four scenarios among 26 LMICs

	Scenario 0(Current trend)	Scenario 1(Modest scale-up)		Scenario 2(Substantial scale-up)		Scenario 3(Universal coverage)	
	MMR (per 100,000 live births)	MMR (per 100,000 live births)	Reduction (%)	MMR (per 100,000 live births)	Reduction (%)	MMR (per 100,000 live births)	Reduction (%)
**Total**	**87**	**79.96**	**7.94**	**62.82**	**27.66**	**52.78**	**39.23**
**Group A**	**170**	**152.24**	**8.98**	**112.03**	**32.42**	**84.90**	**47.77**
Cambodia	224	178.96	19.93	123.11	44.92	83.63	62.58
Indonesia	165	129.94	21.08	91.24	44.59	76.33	53.64
Myanmar	147	132.75	9.79	96.42	34.48	63.58	56.79
Nepal	130	148.72	-14.53	124.28	4.29	122.57	5.61
Papua New Guinea	180	156.98	12.72	121.41	32.49	90.90	49.46
Timor Leste	175	166.06	4.92	115.72	33.74	72.36	58.57
**Group B**	**86**	**82.47**	**-4.11**	**66.43**	**16.27**	**59.41**	**25.61**
Bangladesh	69	107.11	-55.01	90.87	-31.51	79.47	-15.01
Federated States of Micronesia	100	60.50	39.32	50.65	49.20	45.98	53.88
India	82	81.89	0.32	64.32	21.70	55.28	32.71
Kiribati	28	59.82	-113.26	49.68	-77.11	43.48	-55.01
Laos	90	109.79	-22.40	89.69	0.01	75.66	15.65
North Korea	101	87.96	12.87	68.42	32.22	60.35	40.22
Philippines	66	58.39	10.85	39.72	39.36	36.81	43.80
Solomon Islands	108	91.44	15.10	72.46	32.72	66.14	38.59
Tonga	165	104.33	36.60	91.89	44.16	85.74	47.89
Vanuatu	97	63.50	34.50	46.55	51.99	45.21	53.37

**Group C**	**32**	**34.07**	**-10.05**	**29.70**	**3.19**	**26.88**	**12.04**
Bhutan	40	52.02	-31.53	44.49	-12.49	37.94	4.07
China	13	20.98	-65.85	19.41	-53.44	17.89	-41.42
Fiji	35	31.39	10.82	27.29	22.47	23.96	31.93
Malaysia	21	18.78	9.28	16.76	19.03	14.91	27.97
Maldives	61	49.01	19.92	43.23	29.36	43.05	29.66
Mongolia	22	32.31	-46.86	28.98	-31.73	30.29	-37.68
Samoa	59	47.45	20.05	39.67	33.16	34.33	42.16
Sri Lanka	25	25.20	-0.80	21.57	13.72	17.93	28.28
Thailand	25	25.24	0.63	21.92	13.70	19.45	23.43
Viet Nam	33	38.32	-16.12	33.63	-1.91	29.05	11.97

Notes：Maternal mortality ratio (MMR) defined as maternal deaths per 100,000 live births for women of reproductive age (15~49 years). Current trend (Scenario 0), we calculated the MMR in 2030 from the annual percentage change (APC) of 2015~2020. Modest scale-up (Scenario 1), we assumed that coverage of every health intervention increased 2% per year up to a maximum of 100%. Substantial scale-up (Scenario2), we assumed that coverage of every health intervention increased 5% per year up to a maximum of 100%. Universal coverage (Scenario 3), we assumed that coverage of every health intervention reached 95% by 2030. Group A included countries with MMR higher than 140 maternal deaths per 100,000 live births. Group B included countries with MMR from 70 to 140 maternal deaths per 100,000 live births. Group C included countries with MMR less than 70 maternal deaths per 100,000 live births.

Table S4 Additional maternal lives saved by 2030 in three scenarios by group among 26 LMICs

	Group A			Group B			Group C		
	Scenario 1	Scenario 2	Scenario 3	Scenario 1	Scenario 2	Scenario 3	Scenario 1	Scenario 2	Scenario 3
**Periconceptual**	**152**	**361**	**566**	**190**	**461**	**771**	**9**	**17**	**25**
Safe abortion services	152	361	566	190	461	771	9	17	25
**Pregnancy**	**209**	**517**	**1,033**	**253**	**604**	**1,233**	**6**	**14**	**29**
TT - Tetanus toxoid vaccination	3	3	2	20	20	8	0	0	0
Micronutrient supplementation (iron and multiple micronutrients)	3	7	10	14	36	57	0	0	0
Hypertensive disorder case management	203	507	1,021	219	548	1,168	6	14	29
**Childbirth**	**2,224**	**4,028**	**4,420**	**5,123**	**9,220**	**10,613**	**300**	**519**	**714**
Clean birth environment	21	97	96	128	261	216	6	7	5
$MgSO_4$ for eclampsia	274	559	471	305	635	512	40	78	62
Antibiotics for preterm or prolonged PROM	27	50	55	69	137	133	2	5	3
Antibiotics for maternal sepsis	80	151	166	213	413	399	9	14	11
Assisted vaginal delivery	146	342	649	467	1,116	2,242	63	159	311
Uterotonics for postpartum hemorrhage	566	973	737	1,433	2,544	1,858	108	102	55
Manual removal of placenta	114	238	351	286	595	907	17	40	57
Removal of retained products of conception	114	236	357	284	593	926	17	39	62
Cesarean delivery	669	935	710	1,411	1,821	1,281	6	6	1
Blood transfusion	213	447	828	527	1,105	2,139	32	69	147

Notes: Modest scale-up (Scenario 1), we assumed that coverage of every health intervention increased 2% per year up to a maximum of 100%. Substantial scale-up (Scenario 2), we assumed that coverage of every health intervention increased 5% per year up to a maximum of 100%. Universal coverage (Scenario 3), we assumed that coverage of every health intervention reached 95% by 2030. Group A (MMR higher than 140 maternal deaths per 100,000 live births) included Cambodia, Indonesia, Myanmar, Nepal, Papua New Guinea, and Timor Leste. Group B (MMR

from 70 to 140 maternal deaths per 100,000 live births) included Bangladesh, North Korea, India, Kiribati, Laos, Federated States of Micronesia, the Philippines, Solomon Islands, Tonga and Vanuatu. Group C (MMR less than 70 maternal deaths per 100,000 live births) included Bhutan, China, Fiji, Malaysia, Maldives, Mongolia, Samoa, Sri Lanka, Thailand and Viet Nam.

Table S5 Estimated relative reductions on MMR by 2030 under scaling up childbirth intervention 5% per year

	**No scale-up**	**Scaling up childbirth intervention 5% per year**	
	MMR (per 100,000 live births)	MMR (per 100,000 live births)	Reduction (%)
**Total**	**99**	**69.57**	**29.72**
**Group A**	**190**	**126.35**	**33.50**
Cambodia	218	147.49	32.34
Indonesia	173	100.85	41.71
Myanmar	179	113.76	36.45
Nepal	174	129.22	25.74
Papua New Guinea	192	129.51	32.55
Timor Leste	204	137.27	32.71
**Group B**	**103**	**73.67**	**28.41**
Bangladesh	123	94.00	23.58
Federated States of Micronesia	74	55.69	24.74
India	103	67.13	34.83
Kiribati	76	56.03	26.28
Laos	126	94.23	25.21
North Korea	107	70.24	34.36
Philippines	78	45.14	42.13
Solomon Islands	122	89.93	26.29
Tonga	126	103.58	17.79
Vanuatu	94	60.72	35.40
**Group C**	**40**	**31.41**	**21.67**
Bhutan	60	46.16	23.07
China	23	19.41	15.61
Fiji	38	30.71	19.18
Malaysia	21	17.16	18.29
Maldives	57	45.18	20.74
Mongolia	39	29.58	24.15
Samoa	59	44.80	24.07
Sri Lanka	29	23.01	20.66
Thailand	29	23.81	17.90
Viet Nam	46	34.28	25.48

Notes：Maternal mortality ratio (MMR) defined as maternal deaths per 100,000 live births for women of reproductive age (15~49 years). Group A included countries with MMR higher than 140 maternal deaths per 100,000 live births. Group B included countries with MMR from 70 to 140 maternal deaths per 100,000 live births. Group C included countries with MMR less than 70 maternal deaths per 100,000 live births.

Table S6 Estimated MMR in 2030 scaling up intervention coverage 5% per year

	Estimated MMR in 2030
**Cambodia**	
+Safe abortion services	203.38
+Cesarean delivery	181.82
+Uterotonics for postpartum hemorrhage	160.02
+Assisted vaginal delivery	152.67
+Blood transfusion	147.29
+Antibiotics for maternal sepsis	139.77
**Indonesia**	
+Uterotonics for postpartum hemorrhage	148.61
+Cesarean delivery	131.78
**Myanmar**	
+Cesarean delivery	151.88
+Safe abortion services	138.93
**Nepal**	
+Cesarean delivery	159.85
+Uterotonics for postpartum hemorrhage	146.9
+Assisted vaginal delivery	142.57
+Blood transfusion	138.15
**Papua New Guinea**	
+Uterotonics for postpartum hemorrhage	173.49
+Cesarean delivery	162.26
+Blood transfusion	155.84
+Antibiotics for maternal sepsis	148.14
+Assisted vaginal delivery	142.75
+Safe abortion services	137.36
**Timor Leste**	
+Safe abortion services	190.74
+Cesarean delivery	173.06
+Hypertensive disorder case management	166.25
+Assisted vaginal delivery	158.41
+Uterotonics for postpartum hemorrhage	145.69
+Clean birth environment	138.63
**North Korea**	
+Antibiotics for maternal sepsis	93.26
+Assisted vaginal delivery	87.39
+Clean birth environment	84.71
+MgSO4 for eclampsia	80.14
+Uterotonics for postpartum hemorrhage	75.60
+Blood transfusion	73.45
+Hypertensive disorder case management	71.61
+Antibiotics for preterm or prolonged PROM	70.40
+Manual removal of placenta	69.68

**India**	
+Uterotonics for postpartum hemorrhage	89.67
+Cesarean delivery	81.73
+Blood transfusion	78.60
+Assisted vaginal delivery	75.01
+Manual removal of placenta	73.76
+Removal of retained products of conception	72.66
+Antibiotics for maternal sepsis	70.39
+Hypertensive disorder case management	68.98
**Kiribati**	
+Uterotonics for postpartum hemorrhage	69.67
**Federated States of Micronesia**	
+Assisted vaginal delivery	68.29
**Philippines**	
+Cesarean delivery	66.13
**Vanuatu**	
+Uterotonics for postpartum hemorrhage	82.95
+Cesarean delivery	77.91
+Assisted vaginal delivery	74.01
+Blood transfusion	71.67
+MgSO4 for eclampsia	69.37

Notes：Maternal mortality ratio (MMR) defined as maternal deaths per 100,000 live births for women of reproductive age (15~49 years).

Table S7 Estimated relative reductions on MMR by 2030 in four scenarios among 13 LMICs

	Scenario 0 (No scale-up)	Scenario 1 (Modest scale-up)		Scenario 2 (Substantial scale-up)		Scenario 3 (Universal coverage)	
	MMR (per 100,000 live births)	MMR (per 100,000 live births)	Reduction (%)	MMR (per 100,000 live births)	Reduction (%)	MMR (per 100,000 live births)	Reduction (%)
**Total**	**100**	**81.10**	**17.05**	**62.44**	**32.85**	**52.10**	**42.22**
**Group A**	**186**	**147.59**	**20.79**	**108.76**	**41.37**	**86.53**	**52.89**
Cambodia	218	178.96	17.91	123.11	43.53	83.63	61.64
Indonesia	173	129.94	24.89	91.24	47.26	76.33	55.88
Myanmar	179	132.75	25.84	96.42	46.13	63.58	64.48
Nepal	174	148.72	14.53	124.28	28.57	122.57	29.56
**Group B**	**103**	**83.84**	**19.09**	**65.83**	**37.20**	**57.98**	**44.53**
Bangladesh	123	107.11	12.92	90.87	26.12	79.47	35.39
India	103	81.89	20.50	64.32	37.55	55.28	46.33
North Korea	107	87.96	17.79	68.42	36.06	60.35	43.60
Philippines	78	58.39	25.14	39.72	49.08	36.81	52.81
**Group C**	**30**	**25.70**	**12.42**	**22.66**	**22.54**	**19.85**	**31.83**
China	23	20.98	8.78	19.41	15.61	17.89	22.22
Malaysia	21	18.78	10.57	16.76	20.19	14.91	29.00
Sri Lanka	29	25.20	13.10	21.57	25.62	17.93	38.17
Thailand	29	25.24	12.97	21.92	24.41	19.45	32.93
Viet Nam	46	38.32	16.70	33.63	26.89	29.05	36.85

Notes：Maternal mortality ratio (MMR) defined as maternal deaths per 100,000 live births for women of reproductive age (15~49 years). No scale-up (Scenario 0), we assumed that coverage of every health intervention didn't change from baseline. Modest scale-up (Scenario 1), we assumed that coverage of every health intervention increased 2% per year up to a maximum of 100%. Substantial scale-up (Scenario2), we assumed that coverage of every health intervention increased 5% per year up to a maximum of 100%. Universal coverage (Scenario 3), we assumed that coverage of every health intervention reached 95% by 2030. Group A included countries with an MMR in 2020 higher than 140 maternal deaths per 100,000 live births. Group B included countries with an MMR in 2020 from 70 to 140 maternal deaths per 100,000 live births. Group C included countries with an MMR in 2020 less than 70 maternal deaths per 100,000 live births.
